# Supersymmetry-Inspired Non-Hermitian Optical Couplers

**DOI:** 10.1038/srep08568

**Published:** 2015-02-24

**Authors:** Maria Principe, Giuseppe Castaldi, Marco Consales, Andrea Cusano, Vincenzo Galdi

**Affiliations:** 1 Waves Group, Department of Engineering, University of Sannio, I-82100, Benevento, Italy; 2 Optoelectronic Division, Department of Engineering, University of Sannio, I-82100, Benevento, Italy

## Abstract

Supersymmetry has been shown to provide a systematic and effective framework for generating classes of *isospectral* optical structures featuring perfectly-phase-matched modes, with the exception of one (fundamental) mode which can be removed. More recently, this approach has been extended to non-Hermitian scenarios characterized by spatially-modulated distributions of optical loss and gain, in order to allow the removal of higher-order modes as well. In this paper, we apply this approach to the design of non-Hermitian optical couplers with higher-order mode-selection functionalities, with potential applications to mode-division multiplexing in optical links. In particular, we highlight the critical role of the coupling between non-Hermitian optical waveguides, which generally induces a phase transition to a complex eigenspectrum, thereby hindering the targeted mode-selection functionality. With the specific example of an optical coupler that selects the second-order mode of a given waveguide, we illustrate the aforementioned limitations and propose possible strategies to overcome them, bearing in mind the practical feasibility of the gain levels required.

Though seemingly unrelated, classical optics and quantum mechanics exhibit profound formal analogies^1^ essentially stemming from the isomorphism between the Helmholtz equation (governing the vector components of a monochromatic electromagnetic field) and the time-independent Schrödinger equation (describing the wavefunction of a massive particle). Since the early development of quantum theories, these analogies have allowed to share concepts and methods developed in each of the two disciplines, with great mutual benefits in terms of physical understanding and cross-fertilization of ideas. A remarkable example is the “photonic crystal” concept which, translating to photonics certain paradigms and tools originally developed in quantum physics, has enabled unprecedented control of the light flow^2^. In the opposite direction, there is a mounting interest in translating some recent developments in the field of optical metamaterials to the design of novel quantum electron devices^3^.

Recently, new levels of sophistication have been added to the above analogies through the intriguing concepts of “supersymmetry” (SUSY) and “parity-time” (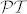) symmetry.

More specifically, the SUSY concept was originally exploited to relate fermions and bosons in string models, and has been subsequently applied to many disciplines, including quantum mechanics, cosmology, as well as disordered and chaotic systems (see Ref. 4 for an introduction and review). In nonrelativistic quantum mechanics, SUSY schemes have been exploited to relate and/or systematically generate classes of Hamiltonians that share the same eigenspectra (with the possible exception of the ground states). More recently, these concepts have been translated to optics^5^ in order to synthesize novel effects and devices, with intriguing applications to quantum cascade lasers^6^, selective mode filtering^7^,^8^ and multiplexing^9^, and transformation-optics^10^, as well as photonic crystals characterized by “invisible defects”^11^ and “one-way invisibility”^12^.

On the other hand, the 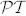-symmetry concept was originally introduced as a possible extension of quantum mechanics (see Ref. 13 for an introduction and review). In spite of the conventional axioms, it was shown that *non-Hermitian* Hamiltonians characterized by complex potentials such that *V*(−*x*) = V*(*x*) (with *x* and * denoting a spatial coordinate and the complex conjugate, respectively) could exhibit *entirely real* eigenspectra. However, beyond some non-Hermiticity threshold in the potential, an abrupt phase transition from a real eigenspectrum (i.e., “exact”, or “unbroken” phase) to a complex eigenspectrum (i.e., “broken” phase) may occur, which is typically referred to as *spontaneous symmetry breaking*^13^. Although the physical validity of such extension of quantum mechanics is an as yet unsettled issue and has recently been challenged^14^, the 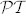-symmetry concept has inspired a wealth of studies in the field of non-Hermitian optics, plasmonics and metamaterials^15^,^16^,^17^,^18^,^19^,^20^,^21^,^22^,^23^,^24^,^25^,^26^,^27^,^28^,^29^,^30^,^31^, aimed at exploring the complex interplay of spatially-modulated optical loss and gain in order to achieve anomalous and otherwise unattainable light-matter interaction effects, including beam switching, unidirectional invisibility, and coherent perfect absorption. Quite remarkably, certain interesting effects may also be attained via mere loss modulation, in completely *passive* configurations^16^,^22^,^29^. Also of interest are certain circuit-based implementations^32^,^33^, which rely on amplifiers to provide the required gain. Finally, worth of mention is also a recent 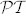-symmetry-based description of optical instabilities in moving media^34^.

Interestingly, the SUSY and 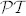-symmetry concepts have also been exploited jointly^8^,^11^,^12^. Of particular interest for what follows is the study by Miri *et al.*^8^, where 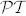-symmetric (and, more in general, non-Hermitian) refractive-index profiles were used in conjunction with SUSY schemes in order to selectively remove certain guided modes in optical waveguides. In particular, starting from a 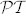-symmetric waveguide in the unbroken phase (i.e., with a real eigenspectrum), a 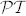-symmetric SUSY partner waveguide was designed so as to exhibit the same eigenvalue spectrum (and, hence, an unbroken phase) apart from a selected mode. It was suggested that this complex extension might relax certain inherent limitations of standard (Hermitian) SUSY schemes^7^, thereby allowing the removal of higher-order guided modes.

These ideas may find disrupting applications to *mode division multiplexing* approaches, which offer a new dimension to increase the capacity of optical links via the capability to manipulate the multiplicity of modes that can propagate in optical waveguides. The key feature in such schemes is an efficient mode (de)multiplexing through a judiciously identification of appropriate procedures for selectively populating and extracting specific modes in an integrated fashion. Originally put forward in connection with optical fibers^35^,^36^, these schemes have proven especially convenient in connection with photonic networks-on-chip, since planar optical waveguides can be fabricated precisely within the chip to accurately control the propagation of guided modes. Within this framework, multiplexers based on weakly guiding asymmetrical Ψ- and *Y* - junctions^37^,^38^,^39^,^40^, as well as multimode interferometers^41^, have been suggested.

Against this background, here we explore the application of SUSY-inspired schemes^8^ to the design of non-Hermitian optical couplers with higher-order mode selection functionalities. Our idea is schematically illustrated in Fig. 1. Starting from a 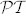-symmetric waveguide, and following the approach in Ref. 8, we design a SUSY-partner waveguide so that the two sets of modes are perfectly phase-matched with the exception of a higher-order mode (the second, in Fig. 1) of the original waveguide. By placing these waveguides in close proximity, the phase-matched modes periodically exchange power (via their exponential tails) between the two structures, and they may be eliminated by suitably inserting a slight loss unbalance in the SUSY-partner waveguide. After a certain distance, this would result in the original waveguide supporting only the mode (*n* = 2, in Fig. 1) that had no counterpart in the SUSY partner waveguide.

It is important to stress that our results are not a direct consequence of the study in Ref. 8, which only proposes a scheme for finding a SUSY-partner of a 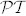-symmetric waveguide, but does not consider the actual coupling between the two structures. Our study here addresses for the first time the coupling between two non-identical 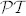-symmetric waveguides, which turns out to play a crucial role in the targeted mode-selection functionality. In particular, via a numerical analysis, we show that, even if the two isolated waveguides are in the unbroken phase (i.e., exhibit real eigenspectra), their coupling generally induces a spontaneous symmetry breaking, i.e., a transition to a complex eigenspectrum. Therefore, straightforward application of the framework in Ref. 8 would not generally yield the desired mode-selection functionality. We show that these limitations can be overcome by resorting to more general (modified-SUSY) classes of isospectral partner waveguides.

## Results

### Review of SUSY formalism

**Figure 1:**
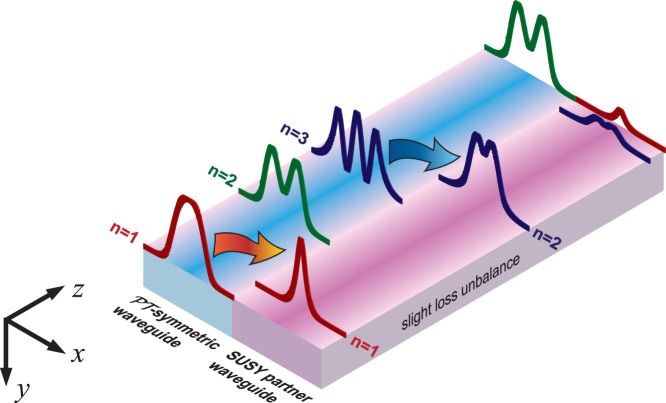
Schematic of a SUSY-inspired non-Hermitian mode-selection system (the structures are assumed to be invariant in the *y* and *z* directions). Starting from a 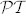-symmetric waveguide in the unbroken phase (i.e., real eigenspectrum, with three propagating modes in this example), a SUSY-partner waveguide is constructed, whose modes are perfectly phase-matched with the original ones with the exception of one modal order (*n* = 2 of the original waveguide, in this example) that is removed [cf. Eqs. (12) and (13)]. By placing in close proximity the two waveguides, the phase-matched modes couple periodically between the two waveguides, and can be filtered out via a slight loss unbalance in the SUSY-partner waveguide. As a result, the original waveguide supports only the *n* = 2 mode.

For the sake of the reader, we compactly review the essentials of the SUSY formalism that are instrumental for our subsequent derivations, referring to Refs. 7, 8 for more details. As schematically illustrated in Fig. 1, we start considering a 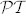-symmetric optical waveguide described by a one-dimensional relative permittivity profile
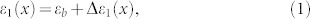
with *ε_b_* denoting a real-valued constant background value, and Δ*ε*_1_ a complex-valued variation satisfying the symmetry condition
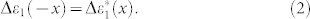
Assuming a transverse-electric (TE) polarization (i.e., *y*-directed electric field) with exp(−*iωt*) time-harmonic dependence, and capitalizing on the translational invariance, the electric field pertaining to a guided mode can be factorized as^42^
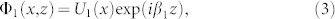
where *β*_1_ is the modal propagation constant, and the dependence on the transverse variable is governed by the Helmholtz equation
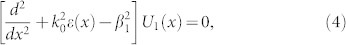
where *k*_0_ = *ω*/*c*_0_ = 2*π*/*λ*_0_ denotes the vacuum wavenumber (with *c*_0_ and *λ*_0_ denoting the corresponding wavespeed and wavelength, respectively). As in Ref. 8, we assume an *unbroken* 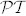-symmetry (i.e., real eigenspectrum), so that regularity-at-infinity conditions are in order. The eigenproblem in Eq. (4) can be compactly recast in an operator form analogous to that utilized in quantum mechanics^7^,
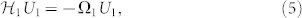
where
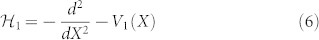
is a Hamiltonian operator,
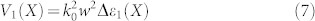
plays the role of a 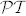-symmetric potential, and
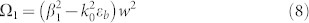
is the (real-valued) eigenvalue, with *X* ≡ *x*/*w* denoting a dimensionless coordinate scaled with respect to a characteristic dimension *w* (e.g., the core width) of the waveguide. Following Miri *et al.*^8^, we aim at constructing a SUSY-partner Hamiltonian,
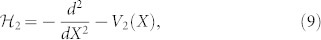
whose eigenvalue spectrum coincides with that in Eq. (6) with the exception of one modal order, say *j*, which is removed. It can be shown (see Ref. 8 for details) that the corresponding potential *V*_2_ is related to the original one in Eq. (7) as follows
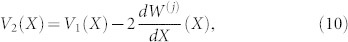
where *W*^(*j*)^ is the “superpotential” associated with the modal order that needs to be removed,
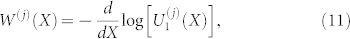
with 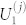 denoting the corresponding eigenfunction. Here an henceforth, the subscripts _1,2_ are used to indicate the original and SUSY-partner configurations, respectively, while the superscript ^(*n*)^ denotes the *n*-th modal order pertaining to either configuration. The eigenspectra of the two configurations are related by^8^
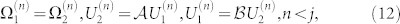

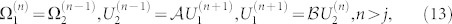
where
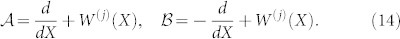
It can be readily verified^8^ that the resulting SUSY-partner Hamiltonian is associated with a 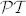-symmetric waveguide in the unbroken phase (i.e., with real eigenspectrum), whose relative permittivity variation immediately follows from the potential in Eq. (10),
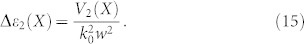
It is important to stress that the non-Hermitian assumption is instrumental for achieving the removal of higher-order modes. For real-valued permittivity profiles (and, hence, potentials), the logarithmic derivative in Eq. (11) would diverge in the presence of nodal points (which always appear in higher-order modes), thereby restricting the applicability of the method to the (nodeless) fundamental mode only.

### Example of mode-selection functionality

**Figure 2:**
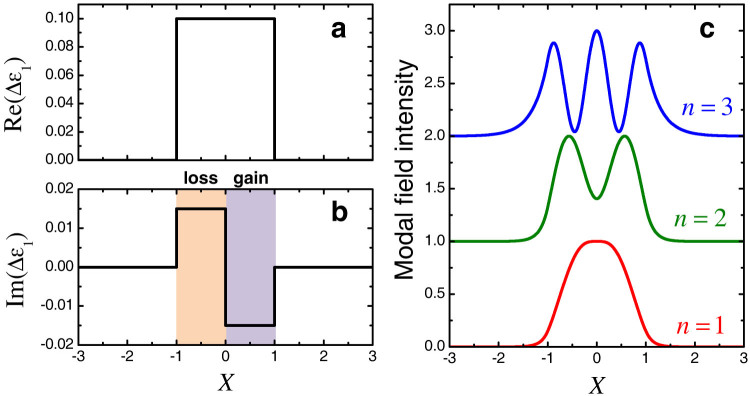
(a), (b) Real and imaginary parts, respectively, of the relative-permittivity variation profile Δ*ε*_1_ [cf. Eq. (1)] pertaining to the original waveguide. Loss and gain regions are highlighted with different shadings. (c) Intensity profiles (vertically offset for clarity) of the corresponding three guided modes (with propagation constants given in Table 1), for *w* = 2*λ*_0_.

Particularizing the above procedure to our scenario in Fig. 1, we start considering a 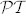-symmetric waveguide with step-index profile, as shown in Figs. 2a and 2b. For such profile, the guided modes can be analytically expressed in terms of complex-argument trigonometric functions, yielding a dispersion equation that generally needs to be solved in the complex plane in order to compute the propagation constants (see Supplementary Information for details). In our specific example, geometric and constitutive parameters are chosen so that the waveguide supports three guided modes, all in the unbroken phase (i.e., real-valued propagation constants), labeled as *n* = 1, 2, 3, in decreasing order of their propagation constants. The intensity profiles of the modal fields are shown in Fig. 2c (note the absence of nodal points), and the corresponding propagation constants are given in Table 1; the phase distributions (not shown for brevity) exhibit odd symmetry, in accord with the unbroken 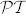-symmetry character.

Particularly critical is the choice of the loss/gain level: on one hand, it should be sufficiently low, so as not to dramatically affect the mode orthogonality, and to maintain all the modes in the unbroken phase. On the other hand, an exceedingly small level of non-Hermiticity in the original waveguide may result in a superpotential [cf. Eq. (11)] with singularities very close to the real axis, and hence unfeasibly high levels of gain in the SUSY-partner waveguide. In our studies below, for simplicity of illustration, we assume *ε_b_* = 1, Re(Δ*ε*_1_) = 0.1 and |Im(Δ*ε*_1_)| = 0.015. While this choice does not directly correspond to a specific material, it allows to illustrate the basic phenomenology in terms of moderately-sized structures (and hence computationally affordable numerical simulations). Nonetheless, we also discuss the implications of different, more realistic choices of material parameters.

**Figure 3:**
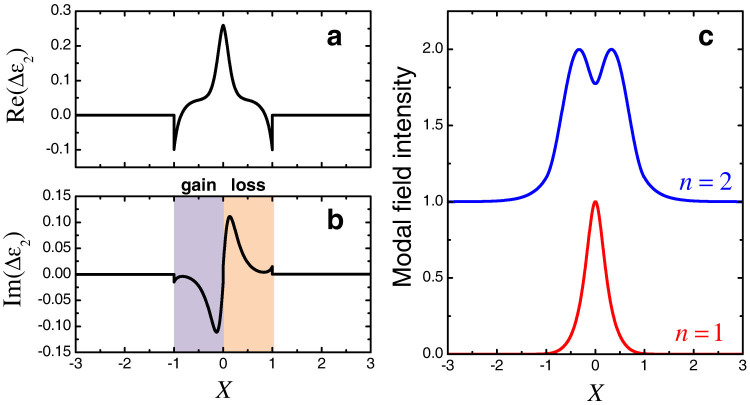
(a), (b) Real and imaginary parts, respectively, of the relative-permittivity variation profile Δ*ε*_2_ pertaining to the SUSY-partner waveguide [cf. Eq. (15)]. (c) Intensity profiles (vertically offset for clarity) of the corresponding two guided modes (with propagation constants given in Table 1), for *w* = 2*λ*_0_.

In order to construct a SUSY-partner waveguide with the same eigenvalue spectrum but without the *n* = 2 eigenvalue, we compute the superpotential *W*^(2)^ [cf. Eq. (11)] and, via Eqs. (10) and (15), we finally obtain the 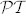-symmetric profile Δ*ε*_2_ shown in Figs. 3a and 3b. As expected, such structure supports only two modes, whose intensity profiles and propagation constants readily follow from Eqs. (12) and (13), and are given in Fig. 3c and Table 1, respectively. As it can be observed, there is no counterpart of the original *n* = 2 modal order.

Paralleling the Hermitian case^7^, one would intuitively expect that, by placing the original waveguide and its SUSY-partner in close proximity, the phase-matched modes would periodically couple between the two structures, and could be filtered out by introducing a slight loss unbalance in the SUSY-partner waveguide, as schematically illustrated in Fig. 1. In what follows, we show that this intuitive picture is generally not valid, and that there are certain critical issues that need to be addressed.

### Modeling aspects

In the Hermitian case^7^, the coupling between the original and SUSY-partner waveguides may be effectively modeled via the coupled-mode theory (CMT)^43^, a variational-based semi-analytical description of the compound structure in terms of the modes of the isolated waveguides. The standard CMT formulation is known to fail in the presence of loss and gain, and has been extended (by judiciously choosing the Lagrangian density and inner product) to *globally* 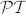-symmetric scenarios in the unbroken phase^44^. However, to the best of our knowledge, there is no CMT extension that is known to work in the presence of more general non-Hermitian scenario like ours, which only exhibits *local* 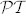-symmetry (as the two waveguides are both 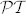-symmetric, but different). We are therefore led to study our structures numerically, via the finite-element-based commercial software COMSOL Multiphysics (see the Methods section below for more details).

### Coupling-induced spontaneous symmetry breaking

**Figure 4:**
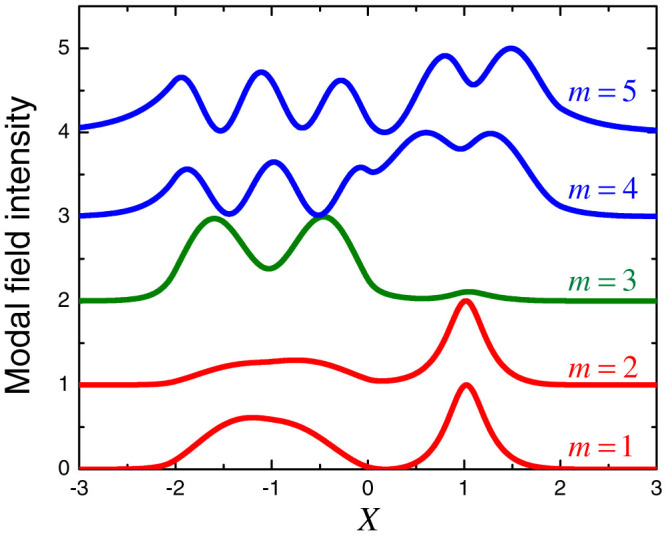
Intensity profiles of the guided supermodes (with propagation constants given in Table 2) of the compound profile in Eq. (16), for *w* = 2*λ*_0_ and *D* = 1.02. The traces are vertically offset for clarity, and different colors are used to facilitate the association with the modes of the isolated waveguides.

We start considering the compound structure described by the profile
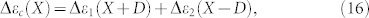
i.e., the juxtaposition of the original and SUSY-partner waveguides, with the normalized distance parameter *D* controlling their coupling. Such structure supports five “supermodes”, with generally *complex-valued* propagation constants. The intensity profiles of the modal fields, for *w* = 2*λ*_0_ and *D* = 1.02, are shown in Fig. 4 (labeled with index *m*), and the corresponding propagation constants are given in Table 2. In particular, the supermodes of order *m* = 1 and *m* = 2 exhibit complex-conjugate propagation constants, whereas the remaining ones essentially exhibit real-valued propagation constants (i.e., imaginary parts that are below our estimated accuracy threshold). Moreover, by comparison with Table 1 and with Figs. 2c and 3c, we observe that the supermodes of order *m* = 1 and *m* = 2 are essentially hybridizations of the *n* = 1 modal orders of the two separate waveguides, with practically identical (real part of) the propagation constants, and with shapes that resemble the combination of these two modes. On the other hand, the supermode of order *m* = 3 resembles in shape and propagation constant the *n* = 2 modal order of the original waveguide, which is missing in the SUSY-partner waveguide, whereas the supermodes of order *m* = 4 and *m* = 5 are essentially hybridizations of the highest-order modes of the two separate waveguides (*n* = 3 in the original, and *n* = 2 in the SUSY-partner).

**Figure 5:**
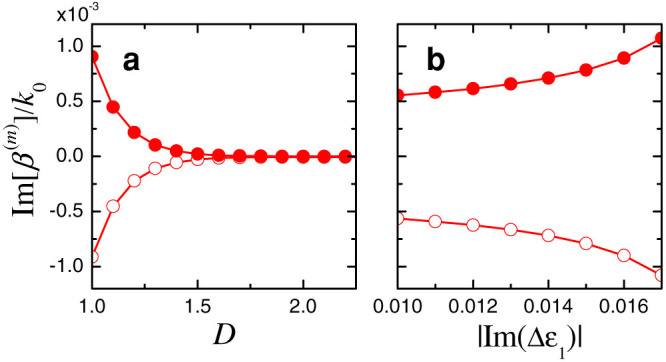
Imaginary parts of the scaled propagation constants *β*^(*m*)^/*k*_0_ pertaining to the supermodes of order *m* = 1 (empty markers) and *m* = 2 (full markers) of the compound profile in Eq. (16), for *w* = 2*λ*_0_, (a) as a function of the normalized distance *D* (for |Im(Δ*ε*_1_)| = 0.015), and (b) as a function of the non-Hermiticity parameter |Im(Δ*ε*_1_)| (for *D* = 1.02).

The above results indicate that, although the two isolated waveguides exhibit unbroken 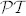-symmetry, the compound structure [cf. Eq. (16)] undergoes a spontaneous symmetry breaking as an effect of the coupling. Figure 5 illustrates this effect by showing the imaginary parts of the propagation constants pertaining to the supermodes of order *m* = 1 and *m* = 2, as a function of the coupling distance *D* and the non-Hermiticity parameter |Im(Δ*ε*_1_)| of the original waveguide. As expected, the imaginary parts asymptotically vanish for large values of the coupling distance (i.e., weakly-interacting waveguides), but are always nonzero and oppositely-signed for finite values of *D* and |Im(Δ*ε*_1_)|. This implies that the desired mode-selection functionality cannot be attained with this configuration, as the exponential amplification of the *m* = 1 supermode would eventually dominate over the coupling effects (see Supplementary Information for more details).

### Modified-SUSY isospectral partners

**Figure 6:**
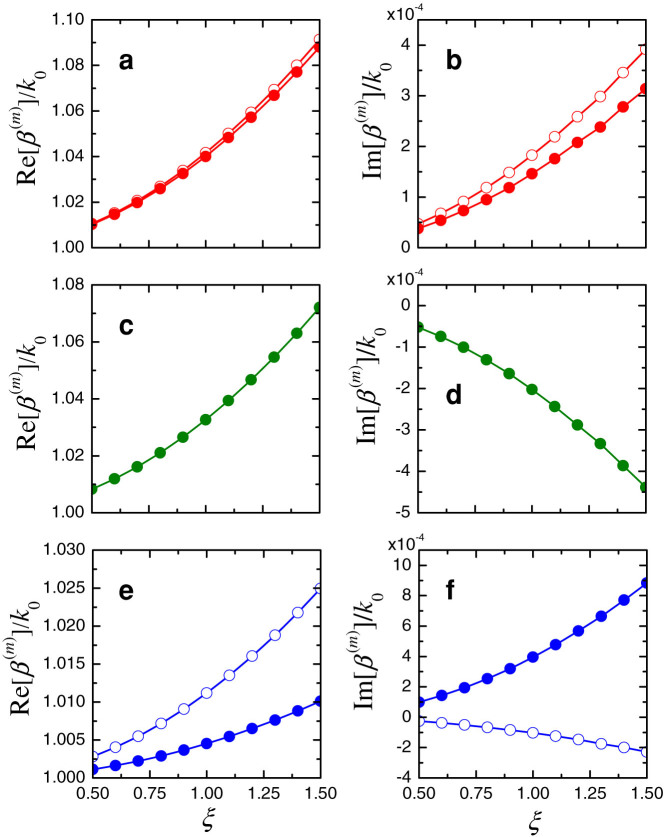
(a), (b) Real and imaginary parts, respectively, of the scaled propagation constants *β*^(*m*)^/*k*_0_ pertaining to the supermodes of order *m* = 1 (empty markers) and *m* = 2 (full markers) of the modified compound profile in Eq. (18), as a function of the scaling parameter *ξ*, for *w* = 2*λ*_0_, *D* = 1.02 and |Im(Δ*ε*_1_)| = 0.015. (c), (d) Same as above, but for the supermode of order *m* = 3. (e), (f) Same as above, but for the supermodes of order *m* = 4 (empty markers) and *m* = 5 (full markers).

The above results indicate that the coupling between two non-Hermitian optical structures generally results in a complex eigenspectrum which may hinder the targeted mode-selection functionality. Nonetheless, this does not necessarily imply that the underlying idea is doomed to failure. In fact, we can show that these limitations can be overcome by generalizing the approach and via judicious exploitation of the available degrees of freedom. First, we note that the SUSY transformation in Eq. (10) is only a particular case of a more general class of Darboux-type transformations^45^, and that more general 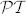-symmetric profiles (e.g., with more than one gain/loss oscillation) for the original waveguide may be considered. It is therefore possible to generate classes of alternative isospectral (apart from a selected mode) partner waveguides whose coupling effects might be compatible with the desired functionality. In an even simpler fashion, maintaining the partner structures in the above example, new isospectral pairs can be easily generated by applying simple spatial transformations to the profiles. The possibly simplest example is perhaps a scaling of the transverse coordinate. For instance, by letting *ξ* > 0 a constant, real-valued scaling factor, it can be readily verified that the modified partner profiles
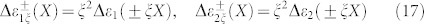
exhibit real-valued eigenspectra, with isospectral properties which differ from those in Eqs. (12) and (13) by a coordinate-scaling. In general, these profiles are no longer SUSY partners in the conventional form, and they will be referred to as “modified-SUSY” isospectral partners. Interestingly, *negative* values of the scaling factor are also meaningful and, in view of the 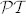-symmetry condition [cf. Eq. (1)], correspond to complex conjugation, i.e., spatial inversion of the loss and gain regions; this introduces a further important degree of freedom. From different combinations of the above profiles, we can generate new, more general classes of non-Hermitian couplers. A particularly interesting example is given by the modified compound profile
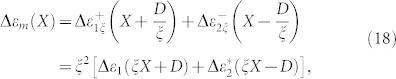
for which a negative scaling (i.e., with complex conjugation) is applied to the SUSY-partner profile. Via a numerical study of the corresponding supermodes as a function of the scaling parameter, it can be observed that the intensity distributions are qualitatively similar to scaled versions of those in Fig. 4 (see Supplementary Information for details), while the propagation constants exhibit a behavior that is markedly different from those in Table 2 and Fig. 5. This is illustrated in Fig. 6, which shows the real and imaginary parts of the supermode propagation constants for the scaling parameter varying within the interval 0.5 ≤ *ξ* ≤ 1.5, and for *D* = 1.02 and |Im (Δ*ε*_1_)| = 0.015. While the real parts only exhibit mild variations, the supermodes of order *m* = 1, *m* = 2 and *m* = 5 now exhibit Im[*β*^(*m*)^] > 0 (i.e., exponential attenuation), and the supermodes of order *m* = 3 and *m* = 4 exhibit Im[*β*^(*m*)^] < 0 (i.e., exponential amplification). These conditions are much more favorable for our targeted mode-selection functionality, as all the undesired supermodes are exponentially decaying, with the exception of the *m* = 4 order (whose amplification is, however, smaller than that associated with the desired *m* = 3 supermode). Interestingly, such conditions are met irrespective of the value of *ξ*, which implies that they inherently stem from the spatial rearrangement of the gain and loss region (i.e., complex conjugation) in the SUSY-related partner waveguide. In fact, it can be verified that the remaining (three) different combinations of the profiles in Eqs. (17) yield configurations that are comparable with (or worse than) the original compound profile in Eq. (16).

**Figure 7:**
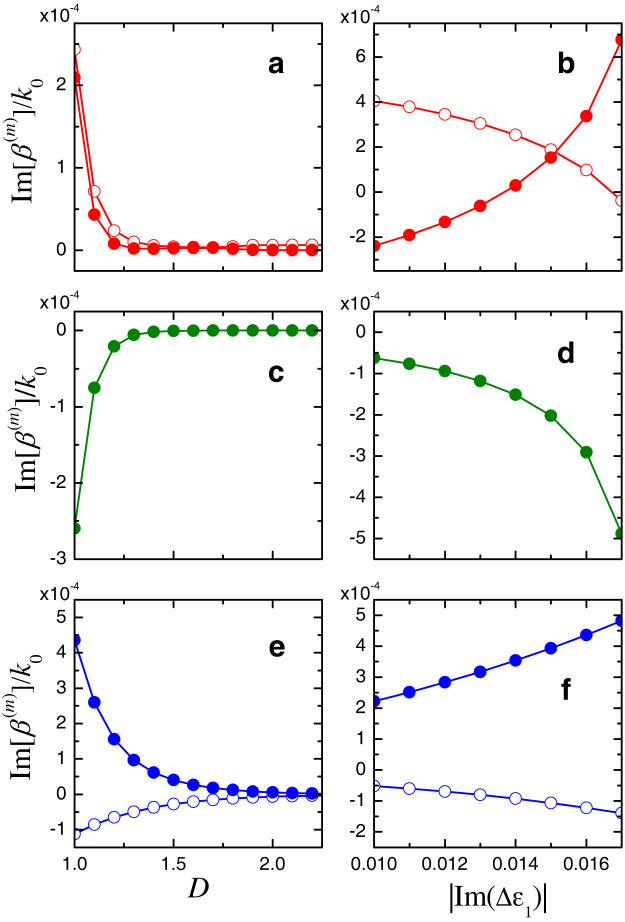
Imaginary parts of the scaled propagation constants *β*^(*m*)^/*k*_0_ pertaining to the supermodes of order *m* = 1 (empty markers) and *m* = 2 (full markers) of the SUSY*-based compound profile in Eq. (19), for *w* = 2*λ*_0_, (a) as a function of the normalized distance *D* (for |Im(Δ*ε*_1_)| = 0.015), and (b) as a function of the non-Hermiticity parameter |Im(Δ*ε*_1_)| (for *D* = 1.02). (c), (d) Same as above, but for the supermode of order *m* = 3. (e), (f) Same as above, but for the supermodes of order *m* = 4 (empty markers) and *m* = 5 (full markers).

For more quantitative assessments, we focus on the particular case of Eq. (18) with *ξ* = 1,
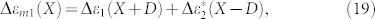
which differs from the original in Eq. (16) only by complex-conjugation of the SUSY-partner profile. For this configuration, henceforth referred to as SUSY*-based, Fig. 7 illustrates the behavior of the imaginary parts of the propagation constants as a function of the coupling distance and the non-Hermiticity parameter, from which observe that the desirable sign inversion in Im [*β*^(1)^] only occurs beyond a critical non-Hermiticity threshold. It this regime, it can be shown (see Supplementary Information for details) that, by introducing a slight loss unbalance *iν* (with *ν* > 0) in the SUSY*-partner waveguide only, it is possible to drive all supermodes in the exponential-attenuation (i.e., Im[*β*^(*m*)^] > 0) regime, with the exception of the desired *m* = 3 supermode which may instead exhibit exponential amplification (i.e., Im[*β*^(3)^] < 0).

**Figure 8:**
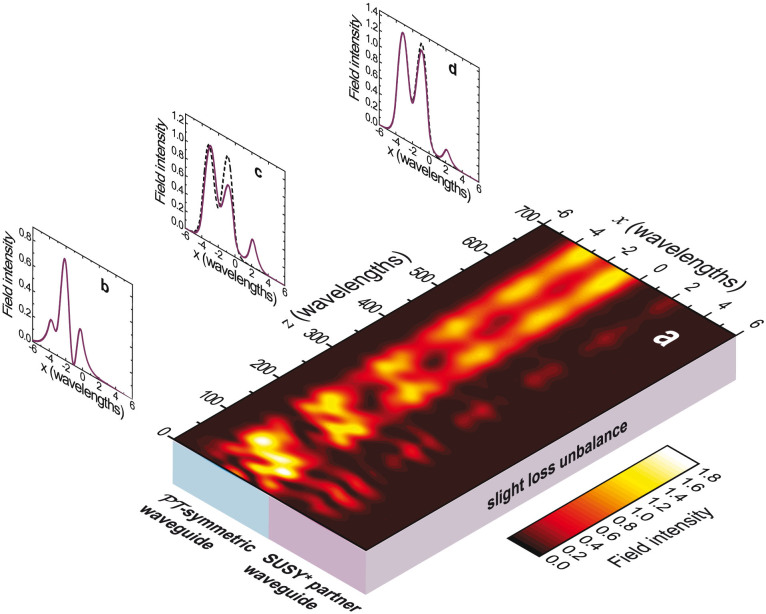
SUSY*-based compound profile in Eq. (19), with *w* = 2*λ*_0_, *D* = 1.02, |Im(Δ*ε*_1_)| = 0.015, and a slight loss unbalance (*ν* = 0.0032) in the SUSY*-partner waveguide. (a) Field-intensity map (in false-color scale) assuming the structure excited by a linear combination of the three guided modes of the original waveguide, with coefficients chosen so that the total power density is equally distributed among the modes. (b), (c), (d) *x*-cuts (continuous curves) for *z* = 0 (input profile), *z* = 335*λ*_0_, and *z* = 700*λ*_0_, respectively. As a reference, also shown (dashed curves) in panels (c) and (d) is the normalized intensity profile of the targeted *n* = 2 mode of the original waveguide.

To better understand these effects, Fig. 8 shows a finite-element-computed intensity field map, and three representative transverse cuts, pertaining to the SUSY*-based compound structure in Eq. (19) (assuming *w* = 2*λ*_0_, *D* = 1.02, |Im (Δ*ε*_1_)| = 0.015, and *ν* = 0.0032) excited with a linear combination of the three modes of the original waveguide (cf. Fig. 2c), with coefficients chosen so that the total power density is equally distributed among the modes. Essentially, the power associated with the *n* = 1 and *n* = 3 original modes periodically oscillates between the two waveguides, with beat lengths that can be roughly estimated (see Supplementary Information for details) as *L_B_* ~ 335*λ*_0_ and *L_B_* ~ 70*λ*_0_, respectively. Since this power couples substantially with the *m* = 1, 2 and *m* = 4, 5 supermodes, respectively, it also decays exponentially. Conversely, the power associated with the *n* = 2 original mode does not couple to the SUSY* partner waveguide, and experiences an exponential amplification. As a result, in qualitatively good accord with our theoretical estimate, the input field distribution (Fig. 8b) is gradually transformed so that at distances comparable with the beat-length *L_B_* ~ 335*λ*_0_ it starts resembling the desired *n* = 2 modal order (Fig. 8c). For larger distances (cf. Fig. 8d), only the modal-order *n* = 2 effectively survives in the original waveguide, thereby attaining the desired mode-selection functionality schematically illustrated in Fig. 1.

## Discussion

To sum up, we have addressed the design of SUSY-inspired non-Hermitian optical couplers with mode-selection functionalities. Our results highlight the crucial role played by the coupling effects. Such effects, not considered in previous studies, need to be taken into account at the design stage since they generally induce a transition to a complex eigenspectrum which may hinder the targeted functionality. With specific reference to a few-mode scenario, we have shown that this phenomenon may be controlled to a certain extent by resorting to modified-SUSY partner profiles characterized by suitable spatial scaling and/or rearrangement of the gain and loss regions. For more complicated configurations requiring the selection of a particularly high-order mode, and hence the tailoring of the complex propagation constant of larger sets of supermodes, further degrees of freedom may be obtained via more complex 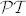-symmetric profiles as well as more general transformations^45^. Within this framework, of particular interest also appear some recently introduced architectures based on optical coupled networks amenable to a discretized Hamiltonian formulation^9^. For these structures, it was hinted in a recent study^9^ that the selection of higher-order modes might be in principle addressed without resorting to loss and gain, although no direct evidence was provided.

Finally, we stress that the specific profiles and parameters in the our case-study are essentially chosen in order to facilitate the illustration of the physical aspects and phenomena, in line with the main scope of this prototype theoretical study. This implies that the structure is not optimized having in mind fabrication-related implications. Nevertheless, we highlight that the required gain values (a few thousand cm^−1^ at near infrared wavelengths, in our example) are in line with the values reported in the literature for semiconductor-based devices^46^,^47^. To give an idea, power gain coefficients of 2000–2600 cm^−1^ and of 6.8 · 10^4^ cm^−1^ have been reported for InGaAs single and double quantum well laser structures^48^ and for a layer of self assembled quantum dots^49^, respectively. This suggests that currently available semiconductor-based gain media may be a viable route for near-infrared implementations. In this case, assuming a more realistic choice 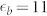 for the background relative permittivity, we can roughly estimate that the dominant beat-length would be *L_B_* ~ 1100*λ*_0_.

Alternatively, we could also reduce the (complex) contrast, so as to tradeoff lower levels of gain with longer structures. For instance, by reducing the contrast of an order of magnitude, say, Δ*ε*_1_ = 0.01 ± *i*0.0015, the dominant beat-length would be *L_B_* ~ 1800*λ*_0_, and the required (maximum) power gain coefficient would decrease to levels ~10^2^ cm^−1^, which are compatible with media made of polymeric matrix and glasses doped with quantum dots or other dopants^50^,^51^,^52^. In all cases, and more likely in this latter scenario, tailoring the spatial concentration of gain ions/dopants can be envisaged as a feasible way to engineer the required gain profiles along the waveguide transverse section^53^.

## Methods

All numerical simulations in our analysis rely on the finite-element-based commercial software package COMSOL Multiphysics.

More specifically, for the study of the supermodes of the compound structures [cf. Eqs. (16), (18) and (19)], we utilize the PDE module to numerically solve the corresponding one-dimensional eigenvalue problems [cf. Eqs. (5)–(8)]. In these simulations, we consider a computational domain of width as large as 60*λ*_0_, discretized with steps of 0.002*λ*_0_, and terminated with Neumann-type boundary conditions.

For the study of the propagation of a given multimode input profile (cf. Fig. 8), we utilize the RF module. In this case, we consider a 12*λ*_0_ × 700*λ*_0_ computational domain, discretized with a maximum mesh-element size of 0.12 *λ*_0_, and terminated by an ad-hoc perfectly-matched layer.

## Author Contributions

M.P., M.C., A.C. and V.G. conceived the idea of this study. M.P. and G.C. developed the theory, designed the structures and carried out the numerical simulations, with inputs and feedback from all the other authors. A.C. and V.G. supervised the project. M.P. and V.G. wrote the manuscript with inputs and revisions from all the other authors.

## Supplementary Material

Supplementary Information Supplementary information

## Figures and Tables

**Table 1:** Scaled propagation constants *β*^(*n*)^/*k*_0_ pertaining to the guided modes of the original waveguide and its SUSY-partner, for *w* = 2*λ*_0_. Here and henceforth, *ε_b_* = 1 is assumed

Mode order	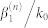	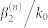
*n* = 1	1.041	1.041
*n* = 2	1.033	1.009
*n* = 3	1.009	-

**Table 2:** Scaled propagation constants (real and imaginary parts) *β*^(*m*)^/*k*_0_ pertaining to the guided supermodes of the compound profile in Eq. (16), for *w* = 2*λ*_0_ and *D* = 1.02. The missing imaginary parts denote computed values below the numerical accuracy threshold (~10^−6^)

Supermode order	Re [*β*^(*m*)^]/*k*_0_	Im [*β*^(*m*)^]/*k*_0_
*m* = 1	1.041	−7.92 × 10^−4^
*m* = 2	1.041	7.92 × 10^−4^
*m* = 3	1.033	-
*m* = 4	1.011	-
*m* = 5	1.004	-
