# Supplementary material for: Supersymmetry-Inspired Non-Hermitian Optical Couplers
Source: Sci Rep. 2015 Feb 24;5:8568. doi: 10.1038/srep08568 (PMC4338421; doi:10.1038/srep08568)
Supplement: Supplementary Information [file srep08568-s1.pdf]

Supplementary Information for: Supersymmetry-Inspired Non-Hermitian Optical Couplers

Maria Principe[1,2], Giuseppe Castaldi[1], Marco Consales[2], Andrea Cusano[*,2] & Vincenzo Galdi[*,1]

[1] *Waves Group, Department of Engineering, University of Sannio, I-82100, Benevento, Italy*

[2] *Optoelectronic Division, Department of Engineering, University of Sannio, I-82100, Benevento, Italy*

**Correspondence and requests for materials should be addressed to A. C. (a.cusano@unisannio.it) or V. G. (vgaldi@unisannio.it).*

Modes of the $\mathcal{PT}$-symmetric step-index waveguide

Assuming a $\mathcal{PT}$-symmetric step-index profile (cf. Figs. 1a and 1b) modeled by Eq. (1) with

$$\Delta\varepsilon_1(x) = \begin{cases} 0, & |x| > w, \\ \varepsilon_a, & -w < x < 0, \\ \varepsilon_a^*, & 0 < x < w, \end{cases} \tag{S1}$$

in the absence of sources, the y-directed electric field of a guided mode can be analytically expressed as

$$E_y(x,z) = \exp(i\beta_1 x) \begin{cases} B_0 \exp(-i\gamma_b x), & x < -w, \\ A_1 \exp(i\gamma_1 x) + B_1 \exp(-i\gamma_1 x), & -w < x < 0, \\ A_2 \exp(i\gamma_1^* x) + B_2 \exp(-i\gamma_1^* x), & 0 < x < w, \\ A_3 \exp(i\gamma_b x), & x > w, \end{cases} \tag{S2}$$

where

$$\gamma_b = \frac{\sqrt{-\Omega_1}}{w} = \sqrt{k_0^2 \varepsilon_b - \beta_1^2}, \quad \text{Im}\left(\gamma_b\right) \geq 0, \tag{S3}$$

$$\gamma_1 = \sqrt{k_0^2 \left(\varepsilon_b + \varepsilon_a\right) - \beta_1^2}, \quad \text{Im}\left(\gamma_1\right) \geq 0, \tag{S4}$$

denote the propagation constants along the x-direction, and $B_0, A_1, B_1, A_2, B_2, A_3$ are unknown expansion coefficients. From Eq. (S2), we can calculate the tangential magnetic field via the appropriate Maxwell's curl equation, viz.,

$$H_z\left(x, z\right) = \frac{1}{ik_0\eta_0}\frac{\partial E_y}{\partial x}\left(x, z\right), \tag{S5}$$

with η_0 denoting the vacuum characteristic impedance. By enforcing the electric and magnetic tangential-field continuity at the three interfaces $x = \pm w$ and $x = 0$, we obtain a 6×6 homogeneous system of linear equations in the unknown expansion coefficients, from which the dispersion relationship follows by zeroing the determinant, viz.,

$$\gamma_1^2\tau_1\left(\gamma_1^* - i\gamma_b\tau_1^*\right) + \gamma_b\gamma_1^*\tau_1\left(\gamma_b - i\gamma_1^*\tau_1^*\right) + 2i\gamma_b\gamma_1^* + \gamma_b^2\tau_1^* + \left(\gamma_1^*\right)^2\tau_1^* = 0, \tag{S6}$$

with

$$\tau_1 = \tan\left(\gamma_1 w\right). \tag{S7}$$

Equation (S6) needs to be generally solved (with respect to γ_b or, equivalently, β_1) in the complex plane. However, for the assumed parameter configurations that maintain the $\mathcal{PT}$-symmetric waveguide in the unbroken phase, real-valued solutions are expected.

Beat-length estimate

The numerical results from the supermode study in the balanced gain/loss scenario can also be utilized to obtain some rough estimates of relevant design parameters, such as the beat-length L_B. Such parameter, defined as the propagation distance for which the power associated with a given mode of the original waveguide has been completely transferred to the partner waveguide, dictates the (minimum) length of the coupler. Our approximate semi-analytical approach is based on projecting the transverse profile $U_1^{(n)}$ of a given mode of the original waveguide onto the set of the numerically-computed supermodes $U_s^{(m)}$, and then using the computed expansion coefficients $c_m^{(n)}$ to propagate the mode, viz.,

$$\Phi_1^{(n)}(x,z) \approx \sum_m c_m^{(n)} U_s^{(m)}(x) \exp\left(i\beta^{(m)} z\right), \tag{S8}$$

with $\beta^{(m)}$ denoting the numerically-computed supermode propagation constants. From Eq. (S8), we can estimate the beat-length by finding the positions of the nulls (or minima) in the field intensity peak distribution along z. Although, in view of the lack of exact orthogonality between the supermodes, this is not strictly rigorous, it still provides a rough estimate in qualitatively good agreement with the finite-element results.

Spontaneous symmetry breaking

Results in Table 2 and Fig. 5 indicate that the supermodes of order $m = 1$ and $m = 2$ pertaining to the compound structure in Eq. (15) are complex conjugate. This implies that the desired mode-selection functionality (cf. Fig. 1) cannot be attained, as the exponential amplification of the

$m = 1$ supermode would eventually dominate over the coupling effects. Figure S1 shows the effects of a slight loss unbalance $i\nu$ (with $\nu > 0$) introduced in the SUSY-partner profile $\Delta\varepsilon_2$, as an attempt to overcome the above limitation. It can be observed that the imaginary part of the propagation constant pertaining to the $m = 1$ supermode (and, hence, its exponential amplification) actually decreases, but this is accompanied by the appearance of positive imaginary parts (and, hence, exponential attenuation) in the propagation constants of the remaining modes. For this configuration, our approximate model in Eq. (S8) predicts that the power associated with the fundamental mode ($n = 1$) of the original waveguide is never significantly transferred to the partner waveguide, and hence a beat-length cannot be meaningfully defined.

To better understand these effects, Fig. S2 shows a finite-element-computed intensity field map, and three representative transverse cuts, pertaining to the compound structure in Eq. (15) (assuming $w = 2\lambda_0$, $D = 1.02$, $|\mathrm{Im}\,(\Delta\varepsilon_1)\,| = 0.015$, and $\nu = 0.008$) excited with a linear combination of the three modes of the original waveguide (cf. Fig. 2c), with coefficients chosen so that the total power density is equally distributed among the modes. As expected, the $m = 1$ supermode profile dominates, resulting in output profiles (at two representative distances) that are quite different from the targeted one (i.e., the $n = 2$ mode of the original waveguide, shown as dashed curves in the transverse cuts).

SUSY*-based configuration

Figure S3 and Table S1 show the numerically-computed intensity profiles and propagation constants, respectively, pertaining to the alternative SUSY*-based compound profile in Eq. (18). As it can be observed, the mode profiles are very similar to the SUSY case (compare with Fig. 4), but the propagation constants are markedly different (compare with Table 2).

Figure S4 shows the effect of a slight loss unbalance in the SUSY* partner profile. We observe that, by properly tailoring this unbalance, it is now possible to drive all undesired supermodes in the exponentially-decaying regime, while maintaining the desired one ($m = 3$) in the exponentially-growing (or unattenuated) regime.

Table S1 | Scaled propagation constants (real and imaginary parts) $\beta^{(m)}/k_0$ pertaining to the guided supermodes of the SUSY*-based compound profile in Eq. (18), for $w = 2\lambda_0$ and $D = 1.02$. The corresponding intensity profiles are shown in Fig. S3.

Supermode order	Re $\left[\beta^{(m)}\right]/k_0$	Im $\left[\beta^{(m)}\right]/k_0$
$m = 1$	1.042	1.88×10^{-4}
$m = 2$	1.040	1.53×10^{-4}
$m = 3$	1.033	-2.02×10^{-4}
$m = 4$	1.011	-1.07×10^{-4}
$m = 5$	1.005	3.93×10^{-4}

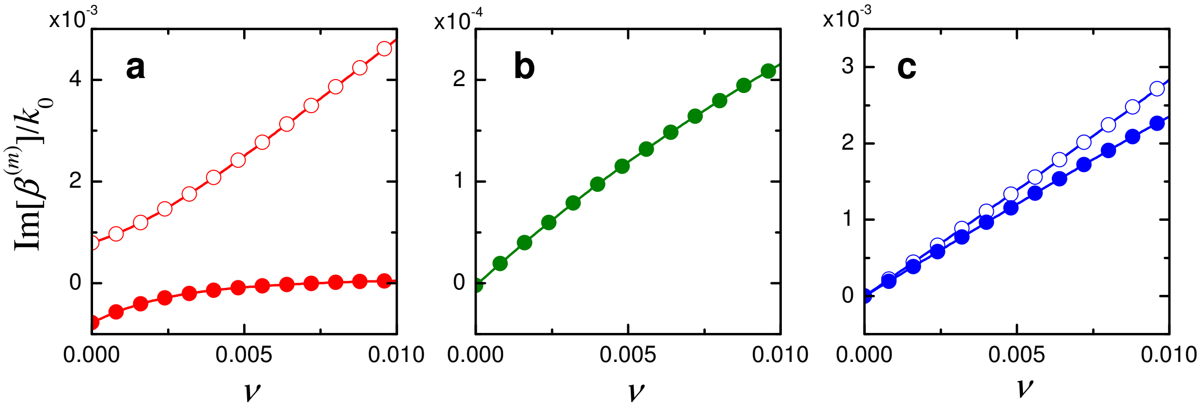

Figure S1 | Imaginary parts of the (scaled) propagation constants $\beta^{(m)}/k_0$ of the supermodes pertaining to the compound profile in Eq. (15), for $w = 2\lambda_0$, $D = 1.02$ and $|\text{Im}(\Delta\varepsilon_1)| = 0.015$, as a function of the loss-unbalance parameter ν in the SUSY-partner waveguide. (a) $m = 1$ (empty markers) and $m = 2$ (full markers); (b) $m = 3$; (c) $m = 4$ (empty markers) and $m = 5$ (full markers).

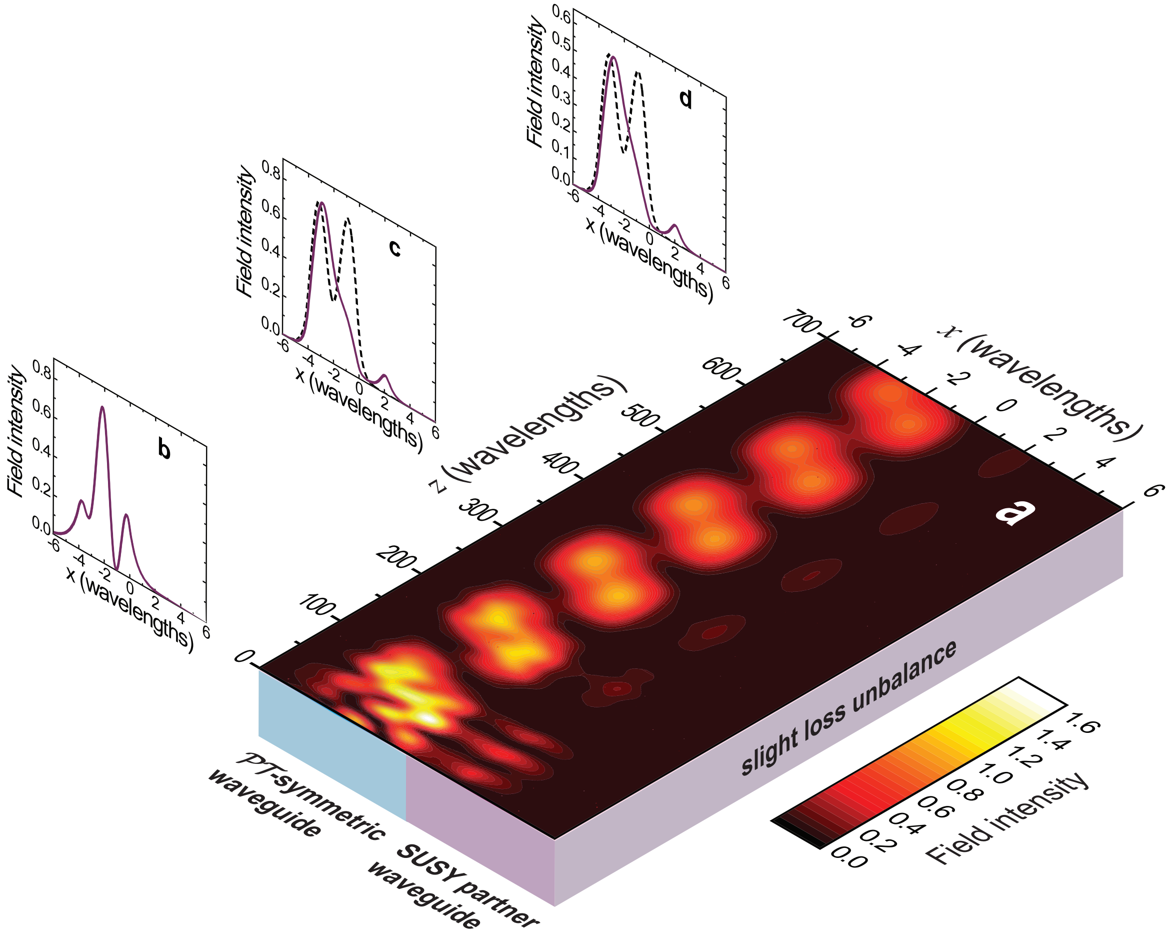

Figure S2 | Compound profile in Eq. (15), with $w = 2\lambda_0$, $D = 1.02$, $|\text{Im}(\Delta\varepsilon_1)| = 0.015$, and a slight loss unbalance ($\nu = 0.008$) in the SUSY-partner waveguide. (a) Field-intensity map (in false-color scale) assuming the structure excited by a linear combination of the three guided modes of the original waveguide (cf. Fig. 2), with with coefficients chosen so that the total power density is equally distributed among the modes. (b), (c), (d) x-cuts (continuous curves) for $z = 0$ (input profile), $z = 335\lambda_0$, and $z = 700\lambda_0$, respectively. As a reference, also shown (dashed curves) in panels (c) and (d) is the normalized intensity profile of the targeted $n = 2$ mode of the original waveguide.

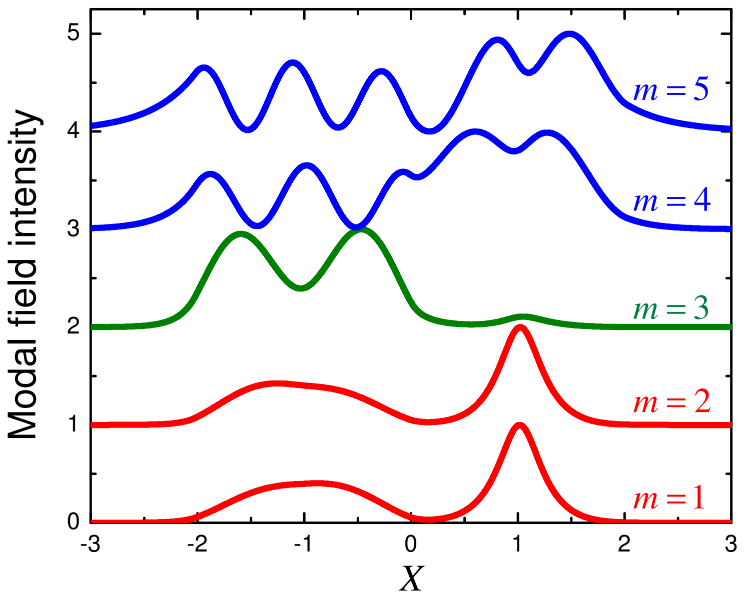

Figure S3 | Intensity profiles of the guided supermodes (with propagation constants given in Table S1) of the SUSY*-based compound profile in Eq. (18), for $w = 2\lambda_0$ and $D = 1.02$. The traces are vertically offset for clarity, and different colors are used to facilitate the association with the modes of the isolated waveguides (cf. Figs. 2c and 3c). Note that the profiles closely resemble those in Fig. 4 pertaining to the compound profile in Eq. (15).

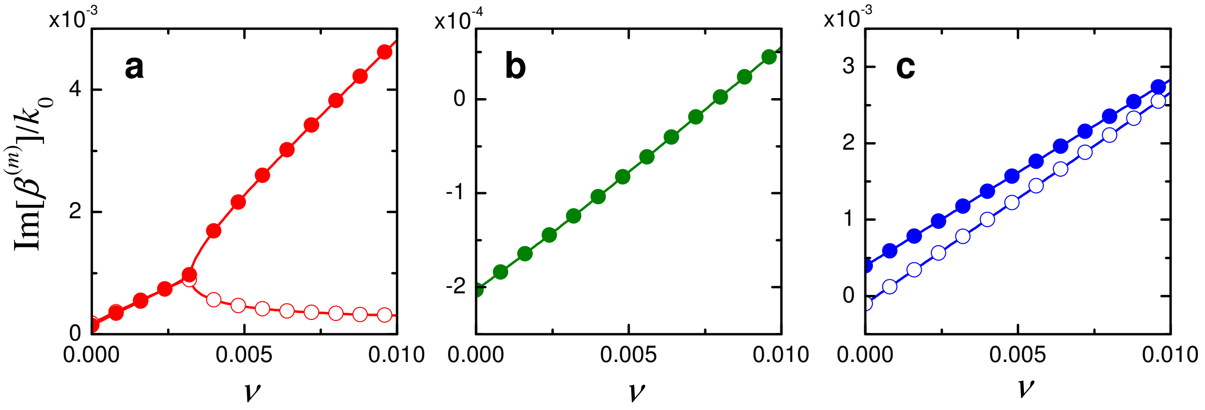

Figure S4 | As in Fig. S1, but considering the SUSY*-based compound profile in Eq. (18).
